# The cerebrospinal fluid in multiple sclerosis: far beyond the bands

**DOI:** 10.1590/S1679-45082017RW3706

**Published:** 2017

**Authors:** Renan Barros Domingues, Gustavo Bruniera Peres Fernandes, Fernando Brunale Vilela de Moura Leite, Charles Peter Tilbery, Rodrigo Barbosa Thomaz, Gisele Sampaio Silva, Cristóvão Luis Pitangueira Mangueira, Carlos Augusto Senne Soares

**Affiliations:** 1 Senne Liquor Diagnóstico, São Paulo, SP, Brazil.; 2 Hospital Israelita Albert Einstein, São Paulo, SP, Brazil.; 3 Faculdade de Ciências Médicas, Santa Casa de São Paulo, São Paulo, SP, Brazil.

**Keywords:** Multiple sclerosis, Cerebrospinal fluid, Biomarkers, Chemokine CXCL13, Fetuins, Neurofilament proteins

## Abstract

The cerebrospinal fluid analysis has been employed for supporting multiple sclerosis diagnosis and ruling out the differential diagnoses. The most classical findings reflect the inflammatory nature of the disease, including mild pleocytosis, mild protein increase, intrathecal synthesis of immunoglobulin G, and, most typically, the presence of oligoclonal bands. In recent years, new biomarkers have emerged in the context of multiple sclerosis. The search for new biomarkers reflect the need of a better evaluation of disease activity, disease progression, and treatment efficiency. A more refined evaluation of disease and therapy status can contribute to better therapeutic choices, particularly in escalation of therapies. This is very relevant taking into account the availability of a greater number of drugs for multiple sclerosis treatment in recent years. In this review, we critically evaluate the current literature regarding the most important cerebrospinal fluid biomarkers in multiple sclerosis. The determination of biomarkers levels, such as chemokine ligand 13, fetuin A, and mainly light neurofilament has shown promising results in the evaluation of this disease, providing information that along with clinical and neuroimaging data may contribute to better therapeutic decisions.

## RESUMO

A análise do líquido cefalorraquidiano tem sido empregada para avaliação diagnóstica da esclerose múltipla e a exclusão dos diagnósticos diferenciais. Os achados clássicos refletem a natureza inflamatória da doença, incluindo discreta pleocitose, leve hiperproteinorraquia, aumento da síntese intratecal de imunoglobulina G e, mais tipicamente, a presença de bandas oligoclonais. Nos últimos anos, surgiram novos biomarcadores para esclerose múltipla, e esta busca por marcadores reflete a necessidade de melhor avaliar a atividade e a progressão da doença, bem como a eficácia terapêutica. Uma avaliação mais refinada da atividade da doença e da resposta aos medicamentos pode contribuir para melhores decisões terapêuticas, particularmente no que se refere à troca de medicação. Isto é muito importante nos dias de hoje, quando surgem novas opções medicamentosas. Neste artigo de revisão, avaliamos criticamente a literatura atual referente aos novos marcadores liquóricos na esclerose múltipla. A mensuração destes marcadores, como a quimiocina CXCL13, fetuína A e, principalmente, o neurofilamento de cadeia leve, demonstrou resultados promissores na avaliação da doença, provendo informações que, em conjunto com dados clínicos e de neuroimagem, podem contribuir para melhores decisões terapêuticas.

## INTRODUCTION

Multiple sclerosis (MS) is an autoimmune disease. Relapsing-remitting multiple sclerosis (RRMS) in the most common form of the disease, in which symptoms resolve spontaneously. After tissue damage accumulates over years, patients often evolve to secondary progressive multiple sclerosis (SPMS), in which neurologic *deficits* gradually worsen over time.^1^ Pathologically, two distinct processes have been identified, namely inflammation/demyelination, which predominates in patients with RRMS; and axonal degeneration, which is considered responsible for SPMS.^2^

The diagnostic criteria of MS are based on clinical and paraclinical assessments emphasizing the need to demonstrate lesions in distinct central nervous system locations (dissemination in space), occurring in separate times (dissemination in time) and to exclude alternative diagnoses. Lesions may be detected either by clinical examination and magnetic resonance imaging (MRI).^3^

The cerebrospinal fluid (CSF) has been studied in for a very long time. The Lange colloidal gold curve, first studied in the diagnosis of syphilis in 1942, showed abnormal protein patterns in patients with MS; however, with low sensibility and specificity.^4^ In the late 1950s, quantitative methods showing an elevated gamma globulin in CSF samples, but not in serum, were introduced.^5,6^ The development of electrophoretic separation of CSF proteins allowed the demonstration of different IgG fractions in the CSF of MS patients. These fractions were first shown in 1959, and were called oligoclonal bands.^7^ The most typical pattern found in MS shows at least two bands in CSF ad none in serum samples.^8^

The sensitivity for detecting oligoclonal bands in MS patients with isoelectric focusing method is approximately 95% and the specificity is greater than 86%. The quantitative IgG index positivity is approximately 75% among oligoclonal bands positive cases.^8^ For some decades, the usefulness of CSF analysis in MS remained restricted to diagnostic evaluation; however, in recent years, many new CSF biomarkers have been studied in MS. These biomarkers are CSF proteins whose CSF concentrations were correlated with clinical and therapeutic parameters in MS. The aim of this study is to critically review the existing literature on the new CSF biomarkers in MS.

## METHODS

A critical review of the literature was conducted on MS and new CSF biomarkers. The search strategy used included the words “multiple sclerosis”, “cerebrospinal fluid”, and “biomarkers” to search PubMed/MEDLINE database, from January 1990 to March 2016.

Each study found was critically analyzed, taking into account the articles referring to biomarkers with greatest potential for incorporation into clinical practice. The study findings were classified as three main axes: CSF biomarkers of disease activity, CSF biomarkers of clinical progression, and CSF biomarkers of therapeutic response.

## CEREBROSPINAL FLUID BIOMARKERS OF DISEASE ACTIVITY

The assessment of disease activity is useful to guide therapeutic interventions. At present, this assessment is based on clinical relapses, disability progression, and the appearance of new lesions in magnetic MRI.^9^ Reliable biomarkers reflecting subclinical disease are still lacking.

### Cytokines and chemokines

Several CSF cytokines and chemokines have been studied as potential markers of MS activity. These molecules participate in the inflammatory response as modulators of cell recruitment and migration to the sites of inflammation. An increase in pro-inflammatory and a decrease in anti-inflammatory cytokines and chemokines occur during MS exacerbations.^10^

Cerebrospinal fluid tumor necrosis factor-alpha (TNF-α) is a pro-inflammatory cytokine. Contradictory results with this biomarker were found in MS. Some investigators described an association between TNF CSF levels and MS activity, but this association was not reproduced by other authors.^11,12^ Interleukin 6 (IL-6)is another pro-inflammatory cytokine, and some studies showed a mild elevation of CSF IL-6 during MS exacerbations.^13,14^ Chemokine ligand 13 (CXCL13) is a B cell chemoattractant that participates in the formation of B cell follicles, which are important in MS pathophysiology. CXCL13 is increased in the CSF of patients with several MS forms and its levels seem to correlate with disease activity.^15,16^

Cerebrospinal fluid CXCL13 appears to be a promising CSF biomarker in the evaluation of MS activity, although the number of studies is still small and the results are not robust enough to propose its use in routine clinical practice.

### Fetuin A

Fetuin A (alpha-2-HS-glycoprotein) is a glycoprotein found by proteomic analysis of CSF. It was associated with increased risk of conversion from clinically isolated syndrome to RRMS.^17^ This glycoprotein is increased in patients with SPMS and also in the CSF of patients with active RRMS, suggesting that elevated fetuin A is potentially useful as a marker of MS activity.^18,19^

### Neurofilaments

Neurofilaments (Nf) are the most important component of the axonal cytoskeleton in neurons and consist of three chains: light (NfL) with 68-70kDa, intermediate (NfI) with 145-160kDa, and heavy (NfH) with 200-220kDa. They provide structural support to neurons and regulate axon diameter. Neurofilaments are released in significant quantity following axonal damage or neuronal degeneration. In these situations, Nf is released into the interstitial fluid and into CSF.^20^ In MS, the CSF NfL concentrations increase after relapses, reaching their peak at 2 weeks after the beginning of symptoms, remaining elevated for at least 15 weeks after an exacerbation.^21,22^ Cerebrospinal fluid NfL levels are associated with the presence of gadolinium enhancing MRI lesions.^23^ These data indicates that increased CSF NfL levels correlate with clinical and MRI parameters, being a potential biomarker of MS clinical activity.

## CEREBROSPINAL FLUID BIOMARKERS OF CLINICAL PROGRESSION

Predicting MS clinical progression remains a great challenge. The difficulty reflects a great heterogeneity of the disease. Axonal degeneration accumulates over the course of the disease and is the primary cause of permanent neurological dysfunction in MS patients. The evaluation of axonal degeneration is based mainly on clinical parameters and MRI findings; however, these parameters are not highly precise. More effective biomarkers of axonal degeneration are still required.^24,25^

### Chemokine ligand 13

The presence of meningeal B-cell follicles is thought to play an important role in clinical progression of MS.^14,26^ One study showed that CXCL13 continuously increases in progressive MS suggesting that CXCL13 correlates with clinical progression.^27^ However, more prospective studies are still necessary to assess the role of CXCL13 in MS prognostic evaluation.

### Light neurofilaments and glial fibrillary acidic protein levels

Cerebrospinal fluid levels reflect neuronal injury and are potentially correlated with axonal degeneration. High CSF NfL levels were positively correlated with higher MS severity score at long-term evolution. Also, higher NfL levels were associated with higher risk of conversion from RRMS to SPMS.^28^ Some studies showed that higher CSF NfL levels were associated with worse long term disability progression. On the contrary, other studies failed to show a correlation between CSF NfL, disease progression, and the risk of conversion to SPMS.^29-31^ Cerebrospinal fluid NfL appears to be a potential biomarker for neuronal damage and clinical progression in MS; nonetheless, more studies are still needed in this area.

Markers of glial damage were also tested in the CSF of MS patients. Glial fibrillary acidic protein (GFAP) is a component of astrocytes filaments. Increased GFAP levels in CSF reflect astrocyte damage. Multiple sclerosis patients have increased CSF GFAP levels when compared to controls. Multiple sclerosis patients with worse ambulation or more severe disability have higher CSF GFAP levels than less disabled patients and controls, suggesting that CSF GFAP may be a biomarker of clinical progression and prognosis.^32-35^

More prospective studies correlating the levels of biomarkers of neuronal and glial degeneration with clinical and neuroimaging data are still necessary to more precisely establish their role in predicting progression of MS.

### Myelin basic protein

Myelin basic protein (MBP) is a component of central nervous system myelin. Myelin basic protein levels in CSF were previously tested as predictor of clinical progression. Cerebrospinal fluid MBP levels increase in acute demyelination; however, high MBP levels did not rise as the disease progressed.^27,36^ Although a potential marker of acute demyelination, MBP does not seem to correlate with MS clinical progression.

## CEREBROSPINAL FLUID BIOMARKERS OF THERAPEUTIC RESPONSE

Several MS therapeutic options have emerged in recent years. Many algorithms have been proposed in order to optimize the use of these drugs, most of them based on the occurrence of clinical relapses, clinical progression, and the appearance of new lesions in MRI. Some new parameters, such as grey matter disease activity and brain atrophy, were also evaluated. However, the parameters to assess the effectiveness of treatment are still imprecise. The contribution of measuring some CSF biomarkers in the assessment of therapeutic efficacy has been studied.^37^

### Light neurofilaments levels

The CSF NfL levels were significantly reduced after immunosuppressive treatment with mitoxantrone.^38^ Cerebrospinal fluid NfL levels were also tested in MS patients treated with natalizumab, with marked decrease during treatment.^31^ Cerebrospinal fluid NfL levels were also studied in patients treated with fingolimod, and reduced levels were observed after the treatment.^39^ Patients treated with fingolimod with no reduction in CSF NfL levels had clinical and MRI deterioration. These findings suggest that at least some MS treatments are associated with a progressive reduction in CSF NfL levels, and that a persistent increase in the levels of CSF NfL may indicate a poor therapeutic response. Further evidence is still needed but the existing data support the use of this biomarker in MS clinical trials.

### Fetuin A

The levels of CSF fetuin A were reduced in patients treated with natalizumab for 1 year.^18,19,40^ More studies with this and other drugs are required to assess the role of this biomarker in MS therapeutic response evaluation.

### Osteopontin

Osteopontin is a matrix protein that works as a pro-inflammatory cytokine. Cerebrospinal fluid osteopontin levels were reduced in patients during natalizumab treatment but it is unknown if these levels are predictive of natalizumab response.^40^ More studies assessing of the role of this biomarker during natalizumab and other MS treatments are still required.

### Chemokine ligand 13

CXCL13 correlates with B-cell activity in MS and it is therefore a good biomarker candidate to evaluate the efficacy of B-cell-targeting therapies, such as rituximab and ocrelizumab. It was shown that CSF CXCL13 was reduced during rituximab therapy; however, its baseline level was not able to predict rituximab response.^41,42^ Other immunossupressive treatments are also able to reduce CXCL13 CSF levels.^38^ Future studies may address if this biomarker predicts therapeutic response, particularly in B-cell-targeting therapies candidates.^43^

## DISCUSSION

The recent studies with new biomarkers indicate that CSF analysis in MS may go beyond diagnosis. Cerebrospinal fluid new biomarkers may contribute in the assessment of disease activity, prognostic evaluation, and therapeutic monitoring of MS.

The existing data suggest that CXCL13, fetuin A, and NfL CSF levels are associated with disease activity. CXCL13, NfL, and GFAP seem to be correlate with prognosis and clinical progression. Neurofilaments intermediate, fetuin A, CXCL13, and osteopontin were associated with therapeutic response, suggesting they can contribute in the assessment of therapy effectiveness.^28^ Therefore, it is possible that in the near future these new CSF biomarkers will contribute to the clinical monitoring of MS patients.

Few points need to be more precisely established. First, it is not known yet how often these markers should be measured. Second, there is not yet a standard clinical interpretation of the results. Finally, a greater standardization in performing these techniques in different laboratories is still required. All these issues should be addressed in future clinical studies in this area.

The better knowledge of new CSF biomarkers levels may refine the assessment of MS patients, supporting clinical decisions that may contribute to more favorable outcomes.

## CONCLUSION

A number of candidate cerebrospinal fluid biomarkers for multiple sclerosis have emerged in recent years. These biomarkers are cerebrospinal fluid proteins that have been shown to correlate with disease activity, disease progression, and treatment response. The future application of such biomarkers measures in clinical practice may provide a more refined and individualized evaluation of the disease, helping optimizing therapeutic decision making.
